# Shall We Abolish the State Medical Association?

**Published:** 1894-09

**Authors:** 


### SHALL WE ABOLISH THE STATE MEDICAL ASSOCIATION?

It seems to us that the impossibility of enrolling even a respectable percentage of the medical profession of the State in a State association, and retaining them, has been abundantly demonstrated, so long as we adhere to our present plan. That it is desired, that it is the hope of the founders to some day claim at least a majority of the regular physicians of the State as members is understood as a matter of course, but the hope can not be realized, in our judgment, without a complete change in the organic laws of the Association. It lacks cohesion. Physicians join, but they do not stick. There are reasons for this, and the defect should be sought and remedied.

In our May issue we pointed out, giving the exact figures and dates, that in 1882 there were 280 members, and that although there were admitted to membership between that date and 1892-3, something over eight hundred physicians, at the Galveston meeting in April, 1893, the roll showed only a membership of 386, or a net gain of 106 members in ten years. We pointed out that, although there were large accessions at each annual meeting, the number was kept down by falling out of those who failed to attend or keep their dues paid up. This is in consequence of the migrations of the body. Those who join at Tyler, say, find it inconvenient to go to Galveston next year, to San Antonio next, and Austin next, and failing to attend, they fail, as a rule, to pay up, and are dropped.

Ten years of failure in the attempt to build up a State association ought to be enough, and we should try another plan—we can but fail, and under the present plan we can never succeed. Yet, to the writer's certain knowledge, the constitution and by-laws have been tinkered with several times; minor defects have been corrected, but without a change, practically, in the organization. Every attempt at a radical change—whereby it conld be hoped to better organize the profession—has been voted down. Dr. T. C. Osborn, at Waco, in 1890, offered a plan of organization by districts, which, in my opinion, would have been a big improvement, but it was received with scant courtesy, and promptly laid on the table.

The fact is, the State is too large and the doctors live too far apart ever to hold them together in an *association by membership*.

THE JOURNAL desires to see the Association absorb all the eligible material in the State and become a power. This we have worked for, but we have concluded that we are working upon a wrong plan, and our efforts are as futile as pouring water in a sieve.

We beg to suggest that Dr. Osborn be invited to again submit his plan of organization by districts, and that it be seriously considered, or else change the plan entirely; endeavor to enroll every physician in some local organization, and let each local society each year send delegates to the *State Medical Convention* at the capital. Do away with members entirely, and hold an annual convention of delegates, representing every section of the State. We earnestly recommend this to the thoughtful consideration of the officers and leading members, and hope they will take some action upon the suggestion at our next meeting. Of course there are many details to be adjusted, and that can be attended to in time. It seems that under a plan of this sort the burden of $5 a year membership, which doubtless drives many out, for they are expected to pay it, whether they attend or not, would be a relief that would be appreciated. In lieu of this, a dollar from each delegate to the Annual State Convention ought to be enough to pay expenses. However, let the suggestion be considered.

In this issue we present the views of Dr. Daniel Parker on the subject. one of our oldest, best known and most respected members. We commend his paper to the thoughtful perusal of our readers.

